# Regulatory interaction between the *ZPBP2-ORMDL3/Zpbp2-Ormdl3* region and the circadian clock

**DOI:** 10.1371/journal.pone.0223212

**Published:** 2019-09-27

**Authors:** Matthew L. Chang, Sanny Moussette, Enrique Gamero-Estevez, José Héctor Gálvez, Victoria Chiwara, Indra R. Gupta, Aimee K. Ryan, Anna K. Naumova

**Affiliations:** 1 The Research Institute of the McGill University Health Centre, Montreal, Quebec, Canada; 2 Department of Human Genetics, McGill University, Montreal, Quebec, Canada; 3 Canadian Centre for Computational Genomics, Montreal, Quebec, Canada; 4 Department of Paediatrics, McGill University, Montreal, Quebec, Canada; 5 Department of Obstetrics and Gynecology, McGill University, Montreal, Quebec, Canada; University of Lübeck, GERMANY

## Abstract

Genome-wide association study (GWAS) loci for several immunity-mediated diseases (early onset asthma, inflammatory bowel disease (IBD), primary biliary cholangitis, and rheumatoid arthritis) map to chromosomal region 17q12-q21. The predominant view is that association between 17q12-q21 alleles and increased risk of developing asthma or IBD is due to regulatory variants. ORM sphingolipid biosynthesis regulator (*ORMDL3*) residing in this region is the most promising gene candidate for explaining association with disease. However, the relationship between 17q12-q21 alleles and disease is complex suggesting contributions from other factors, such as *trans*-acting genetic and environmental modifiers or circadian rhythms. Circadian rhythms regulate expression levels of thousands of genes and their dysregulation is implicated in the etiology of several common chronic inflammatory diseases. However, their role in the regulation of the 17q12-q21 genes has not been investigated. Moreover, the core clock gene nuclear receptor subfamily 1, group D, member 1 (*NR1D1*) resides about 200 kb distal to the GWAS region. We hypothesized that circadian rhythms influenced gene expression levels in 17q12-q21 region and conversely, regulatory elements in this region influenced transcription of the core clock gene *NR1D1* in *cis*. To test these hypotheses, we examined the diurnal expression profiles of *zona pellucida* binding protein 2 (*ZPBP2/Zpbp2*), gasdermin B (*GSDMB*), and *ORMDL3/Ormdl3* in human and mouse tissues and analyzed the impact of genetic variation in the *ZPBP2/Zpbp2* region on *NR1D1/Nr1d1* expression. We found that *Ormdl3* and *Zpbp2* were controlled by the circadian clock in a tissue-specific fashion. We also report that deletion of the *Zpbp2* region altered the expression profile of *Nr1d1* in lungs and ileum in a time-dependent manner. In liver, the deletion was associated with enhanced expression of *Ormdl3*. We provide the first evidence that disease-associated genes *Zpbp2* and *Ormdl3* are regulated by circadian rhythms and the *Zpbp2* region influences expression of the core clock gene *Nr1d1*.

## Introduction

Human chromosomal region 17q12-q21 harbors risk alleles for several immunity-mediated diseases, including early onset asthma, inflammatory bowel disease (IBD), primary biliary cholangitis (PBC), and rheumatoid arthritis (RA) [1–7]. This suggests that the disease-associated variants (daVs) in this region are likely to have pleiotropic effects and impact a pathway(s) that is critical for the pathogenesis of not one, but several immunity-mediated diseases (reviewed in [8]). Identification of the gene responsible for the genetic association with disease is complicated by the fact that no polymorphisms that would alter the protein sequence are found in the best gene candidate ORM sphingolipid biosynthesis regulator (*ORMDL3*) whereas regulatory genetic variants influence expression of at least three protein-coding genes residing in this region, *zona pellucida* binding protein 2 (*ZPBP2*), gasdermin B (*GSDMB*) and *ORMDL3*, suggesting that transcription of these genes is governed by shared regulatory mechanisms [1, 9, 10]. Our search for functional genetic *cis*-regulatory variants identified several candidates, the best characterized of which is rs12936231-C/G located in intron 5 of *ZPBP2* [10, 11]. It causes loss/gain of the insulator protein CTCC factor (CTCF) binding site [10–12]. Allelic differences in CTCF-binding lead to differences in chromosome conformation [10, 13]. Therefore, it has been proposed that loss of CTCF site (the rs12936231-C allele) in the *ZPBP2* gene resulted in chromatin conformation that favored interaction between the promoters of *ORMDL3* and *GSDMB* and a distant enhancer leading to higher transcription rates [10, 13]. Of the three genes, *ORMDL3* has been singled out as the best candidate for explaining the link between the 17q21 alleles and asthma due to its strong and highly reproducible eQTL, higher expression levels in asthmatic subjects and its role in sphingolipid metabolism, which has recently come to the fore as a significant modulator of lung hypersensitivity and inflammatory responses [14, 15]. However, the association between lipid levels and the *ORMDL3* genotype in humans remains unclear [16–18]. Moreover, conflicting evidence from different mouse studies puts into question the causal relationship between *Ormdl3* and asthma [19–22]. Thus, the sum of current data suggests a complex relationship between the 17q12-q21 daVs and disease, such as a cumulative effect from variation in expression of several neighboring genes, impact of modifier loci, epigenetic variation, environmental factors, or an association that is time-dependent. Here, we explored the latter possibility.

The circadian clock system regulates multiple biological processes from homeostasis, metabolism, and immunity to reproduction and behavior (reviewed in [23, 24]). The molecular circadian clock consists of several core clock genes that drive 24h oscillations in the expression of thousands of genes throughout the genome. Mutations in core clock genes lead to phenotypic abnormalities in mice, whereas in humans, factors that disturb circadian rhythms, such as jet lag or shift work, are suspected to increase the risk of developing certain immune-mediated diseases (reviewed in [25]). Remarkably, the core clock gene nuclear receptor subfamily 1, group D, member 1 (*NR1D1*, also known as REV-ERB-alpha) resides about 200 kb distal to the top asthma and IBD daVs in chromosomal region 17q21. NR1D1 is a transcription factor that regulates thousands of genes throughout the genome and links the circadian clock with lipid metabolism [26, 27]. Evidence from chromatin conformation studies suggests that the *ZPBP2*-*ORMDL3* and *NR1D1* regions physically interact [28]. However, whether the *ZPBP2-ORMDL3* daVs modify the diurnal expression profile of *NR1D1* remains unknown.

To gain better understanding of the regulatory mechanisms governing gene expression in the 17q12-q21 disease-associated region, we asked two questions: 1) whether the 17q12-q21 disease gene candidates were regulated by circadian rhythms; and 2) whether regulatory elements in the *ZPBP2* region influenced transcriptional regulation of *NR1D1*.

First, using public databases we examined diurnal fluctuations in the expression levels of *ZPBP2*, *ORMDL3* and *GSDMB* and the mouse orthologs *Zpbp2* and *Ormdl3* and found diurnal rhythms in the mouse genes. Second, we tested the hypothesis that the *ZPBP2* region harbored regulatory elements that influenced expression of *NR1D1* using publicly available data from human lymphoblastoid cell lines. Third, we tested the same hypothesis by comparing expression profiles of *Nr1d1* in wild type (WT) and *Zpbp2* mutant mice (KO) that carry a deletion of the 5’ portion of the *Zpbp2* gene including its promoter and enhancer regions. We find that the *Zpbp2* deletion influences diurnal regulation of *Nr1d1* in lungs and ileum. We also show that the deletion is associated with enhanced expression of *Ormdl3* in liver, supporting previous findings that implicate the human *ZPBP2* region in transcriptional regulation of *ORMDL3*.

## Materials and methods

### Mice

The B6.129S7-*Zpbp2*^tmZuk^/Mmjax (MMRRC#42297) strain of mice [29] that carry a deletion of the 5’ region of *Zpbp2* (exons 1 to 3) [30] in a C57BL/6J genetic background (referred to as KO from this point on) was maintained in our mouse facility for several years. Control C57BL/6J mice (WT) were born and maintained in the same colony as KO mice. All mice were housed in 12h:12h light/dark cycle and had access to food and water *ad libitum*. Lungs, liver and small intestine (ileum) from adult KO and WT mice of both sexes were collected every 4 hours (Zeitgeber times ZT2, ZT6, ZT10, ZT14, ZT18, and ZT22) over a 24 h period (n = 3 mice per time point/genotype). Between ZT12 and ZT0 (dark phase) all procedures including organ harvesting were performed under red light. To validate the effect of genotype on expression, independent sets of ZT10 samples from mice of both genotypes and both sexes were assayed (n = 4–7 for WT mice, n = 5–10 for KO mice). All procedures were conducted in accordance with the guidelines set by the Canadian Council of Animal Care (Ottawa, Ontario, Canada) and were approved by the Animal Care Committee of the McGill University Health Center (Montreal, Quebec, Canada).

### Expression analysis

RNA was extracted, cDNA synthesized, and expression levels assayed in each mouse sample individually. RNA extraction, cDNA synthesis, and analysis of gene expression levels were performed as previously described [29]. Expression levels of *Nr1d1*, *Ormdl3* and *Zpbp2* were determined in both WT and KO mice using RT-qPCR and normalized to the levels of eukaryotic translation elongation factor 2 (*Eef2*) RNA. The reliability of *Eef2* as a normalization control has been validated in our previous study [29]. For diurnal expression profiling, the peak level of average expression in WT mice was set at 1.0. For the validation experiments, the mean of WT expression was set at 1. Primers are listed in S1 Table.

#### RNA-sequencing

Two independent RNA-sequencing experiments were conducted on RNA from lungs. Lungs were collected from 3 *Zpbp2* KO (KO1-KO3) and 3 WT (WT1-WT3) mice at ZT7 and 3 *Zpbp2* KO (KO4-KO6) and 3 WT (WT4-WT6) mice at ZT10. The sequencing for both experiments was done at the McGill University and Genome Quebec Innovation Centre (Montreal, QC, Canada). Differential gene expression analysis was performed using the GenPipes RNA-seq pipeline [31]. Briefly, reads were trimmed and filtered for quality, then they were aligned to the mouse reference genome (GRCm38) using STAR [32]. The abundance of each transcript was estimated using HT-Seq Count [33]. Differential gene expression was determined with both the EdgeR [34] and DESeq [35] packages. Pathway analysis was done using DAVID Bioinformatics Resources 6.8 software [36].

#### Use of publicly available expression data

Publicly available expression data were used to assess diurnal or circadian variation in the transcription of human 17q12-q21 genes or their mouse orthologs, respectively. Human expression data from adipose tissue (GSE87761 [37]), skin biopsies (GSE112660 [38]), peripheral blood cells (GSE107537 [39] and GSE48113, [40]) were used to examine diurnal variation in *ZPBP2*, *ORMDL3* and *GSDMB*. To determine whether expression of mouse genes *Ormdl3* and *Zpbp2* was regulated by circadian rhythms in nine different peripheral tissues, expression data from the circadian gene expression atlas assayed by microarray (GSE54652, C57BL/6J male mice kept in constant darkness (DD) [41]), liver from C57BL/6J male mice maintained in light/dark conditions (GSE52333, [42]), distal colon from male mice maintained in light/dark conditions (GSE10644 [43]) were used.

To determine the effect of *ZPBP2* genotype on *NR1D1* expression levels, expression data for human genes *ORMDL3*, *GSDMB*, *ZPBP2*, and *NR1D1* were obtained from the Gene Expression Omnibus (GEO) datasets for lymphoblastoid cell lines (GSE8052 [1]).

### Statistical analyses

Comparisons between groups were done using the GraphPad Prizm 8.0 software (one-way or two-way ANOVA followed by post hoc analyses with Sidak’s correction for multiple testing). Effect sizes were calculated using Cohen’s ***d*** [44] (http://www.socscistatistics.com). The Jonckheere-Terpstra-Kendall (JTK) p-values for mouse circadian expression data were taken from the Circadian Expression Profiles Database (CircaDB at http://circadb.hogeneschlab.org).

## Results

### Diurnal/Circadian regulation of 17q12-q21 genes and their mouse orthologs

**Fig 1:**
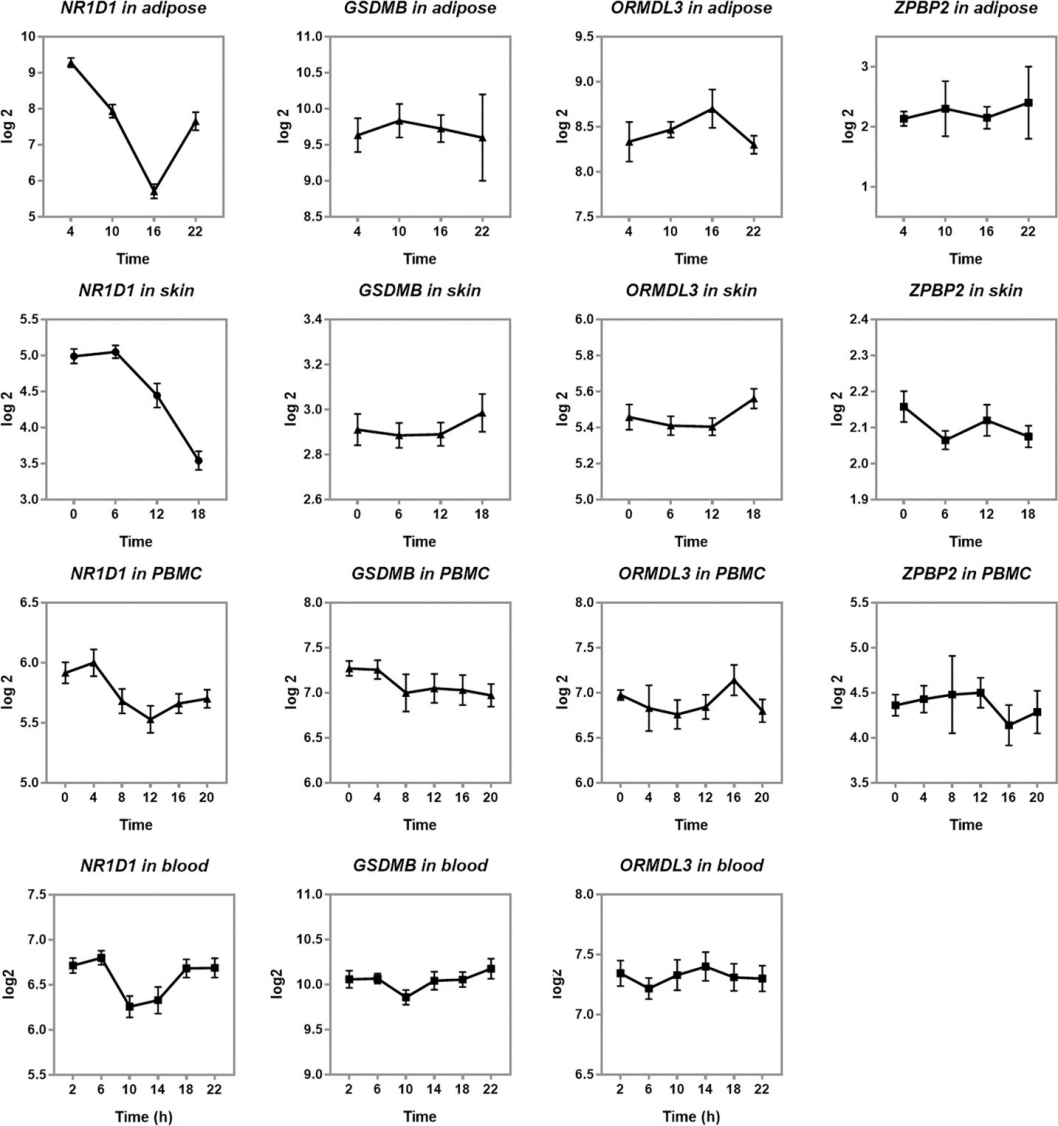
Diurnal profiles of *NR1D1*, *ORMDL3*, *GSDMB* and *ZPBP2* in human adipose tissue (n = 3), skin (n = 20), peripheral blood mononuclear cells (PBMC) (n = 11), and blood from individuals where circadian rhythms were synchronized with melatonin levels (n = 6–11) (data extracted from GSE87761, GSE112660, GSE107537, and GSE48113, respectively). The y-axis shows log2 of relative expression levels, the x-axis shows time of the day. *ZPBP2* expression data were not available for GSE48113.

To test the hypothesis that *ZPBP2*, *GSDMB*, or *ORMDL3* were regulated by circadian rhythms in human tissues, we extracted gene-specific data from expression datasets with sample collection covering 24h time periods. Diurnal oscillations in RNA levels were observed for core clock gene *NR1D1* (Fig 1). We found no statistically significant effect of time of the day on regulation of *ORMDL3*, *ZPBP2*, or *GSDMB*. Nevertheless, it is worth noting that *ORMDL3* tended to have higher expression levels in the afternoon when *NR1D1* levels were at their minimum.

Circadian regulation varies between different cell types and human datasets that include time of sample collection are limited to tissues that are easily obtainable from live donors. To get a better idea of the circadian as well as tissue-specific variation in the expression of our genes of interest, we examined expression data from mice that were kept in constant darkness [41]. The mouse orthologs *Zpbp2*, *Ormdl3*, and *Nr1d1* reside on chromosome 11 with most of the region being conserved. However, the mouse lacks a transcribed *GSDMB* gene ortholog between *Zpbp2* and *Ormdl3*.

**Fig 2:**
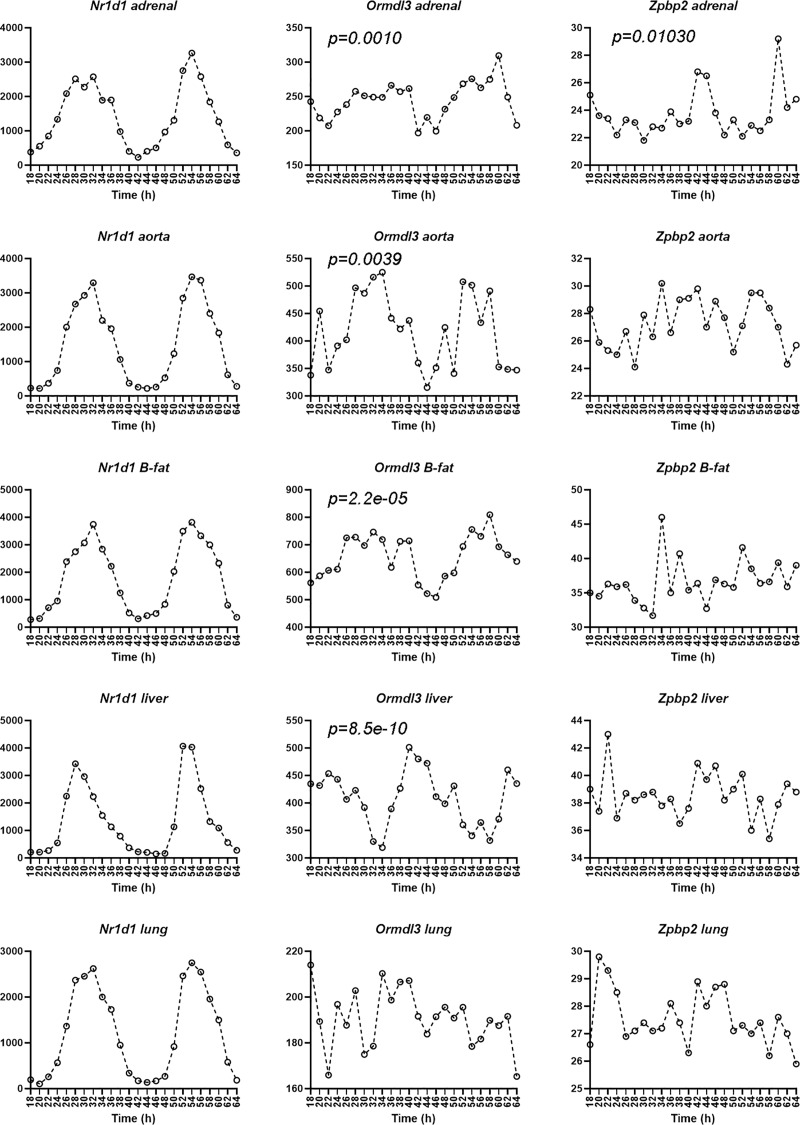
*Ormdl3* and *Zpbp2* are regulated by the circadian clock in mouse organs. Circadian oscillations of *Ormdl3* or *Zpbp2* RNA levels in adrenal, aorta, brown adipose tissue (B-fat), liver, and lungs of wild type C57BL/6J male mice. The x-axis shows time since beginning of DD in hours. The y-axis shows expression levels measured on the individual gene chips. Data extracted from GSE54650 [41]. JTK p-values are taken from the Circadian Expression Profiles Database.

Data from nine peripheral organs (e.g. adrenal, aorta, brown adipose tissue, heart, kidney, liver, lung, skeletal muscle, and white adipose tissue (GSE54650 [41]) from mice kept in constant darkness and livers and distal colon from mice maintained in light/dark cycle ([42, 43]) were examined to determine if *Ormdl3* or *Zpbp2* transcription was controlled by the circadian clock and if the circadian regulation was tissue-specific. *Ormdl3* showed circadian oscillations in expression in the adrenal, aorta, brown adipose tissue, and liver, but not lungs, kidney, white adipose tissue, skeletal muscle, or heart (Fig 2 and data from the Circadian Expression Profiles Database, [41]). *Zpbp2* levels oscillated in the adrenal (Fig 2). *Nr1d1* levels showed circadian oscillations in all peripheral organs, as expected (Fig 2). It is worth noting that mice are nocturnal animals and the circadian rhythm of mouse *Nr1d1* expression is shifted compared to the human *NR1D1*, which peaks in the early morning and has a trough during the day (Fig 2). Interestingly, *Ormdl3* expression in adrenal, aorta, and brown adipose tissue peaked during the subjective day (see figure legend for detail), whereas in liver it peaked during the subjective night (Fig 2). *Zpbp2* expression in adrenal peaked during the subjective night when *Nr1d1* expression was at its trough.

Diurnal oscillations in *Nr1d1*, *Ormdl3* or *Zpbp2* levels were also detected in mice maintained in light/dark conditions (S1 Fig) [42, 43]. Thus, analysis of published and publicly available data demonstrates that *Ormdl3* and *Zpbp2* are controlled by the circadian clock in the mouse and this regulation is tissue-specific.

### *Cis*-regulatory effects on *NR1D1* expression levels in human LCLs

**Fig 3:**
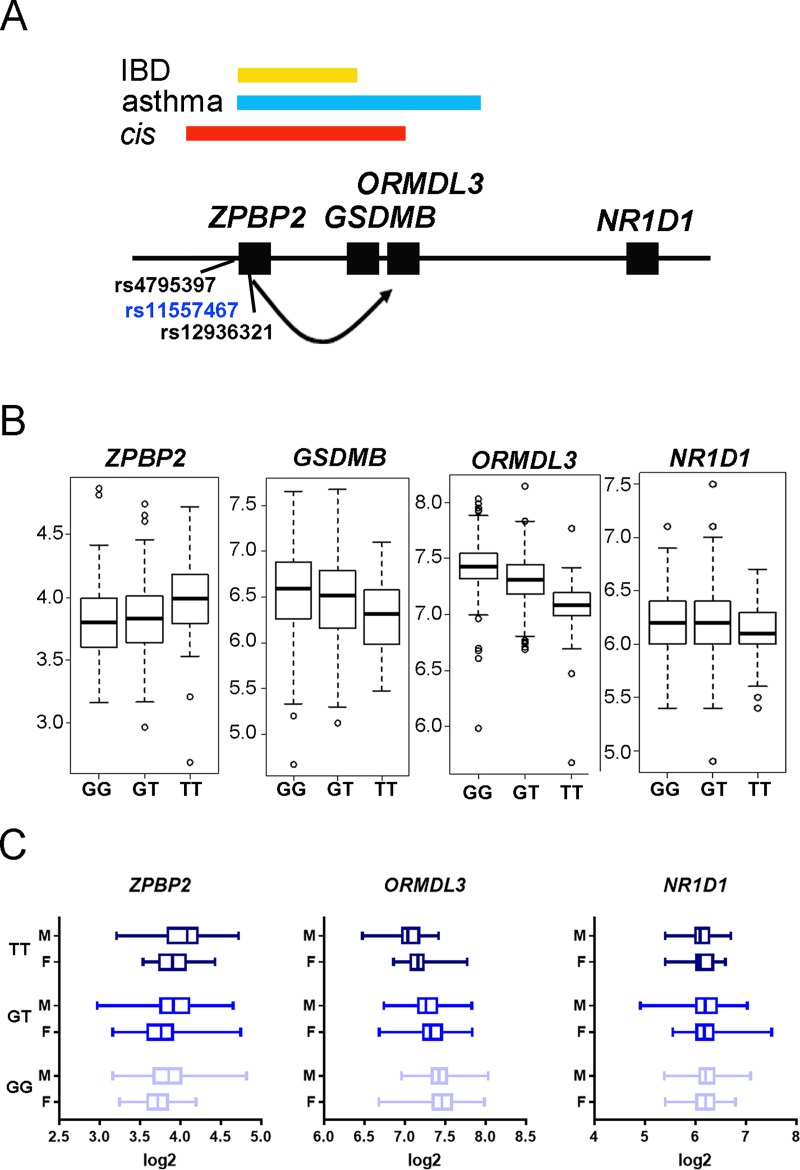
Genetic variation in the *ZPBP2* region is associated with variation in expression levels of *ORMDL3*, *GSDMB*, *and ZPBP2* in human cells. **A. The 17q12-q21 region is associated with several common complex genetic diseases** (schematic representation, not in scale). Regions associated with asthma, IBD, and the *cis*-regulatory region for *GSDMB* and *ORMDL3* are shown. Genes are represented by filled squares. Functional *cis-*regulatory SNPs associated with *ZPBP2*, *ORMDL3* and *GSDMB* transcription levels are located in the *ZPBP2* gene region. **B. The rs11557467 genotype is associated with variation in 17q12-q21 gene expression levels in human LCLs**. The x-axis shows genotype of the LCLs, the y-axis shows log2 of relative RNA levels. **C. Sex of the donor influences expression levels of *ZPBP2* and *ORMDL3*, but not *NR1D1*.** The x-axis shows log2 of relative RNA levels, the y-axis shows genotype and sex of the donor. Data from 336 LCLs (119 GG: 45 females, 74 males; 163 GT: 89 females, 74 males; 54 TT: 16 females, 38 males).

Genetic variation in the *ZPBP2* gene region is associated with variation in the expression levels of *ZPBP2*, *GSDMB*, and *ORMDL3* in *cis* [1, 9, 10]. To test the hypothesis that the same regulatory variants also influenced expression of *NR1D1*, we analyzed publicly available expression data from 336 EBV-transformed LCLs derived from a familial cohort of asthmatic and non-asthmatic children from the UK [1]. Genotypes for functional SNPs rs4795397 and rs12936231 were not available in this cohort. Instead, we used genotype data for a closely linked SNP rs11557467-G/T located in exon 4 of *ZPBP2* less than 500 bp proximal to rs12936321 (Fig 3A). We noticed that sex ratios varied between genotype groups (sex ratios of 1.6 for GG, 0.8 for GT and 2.3 for TT). To make sure that sex bias did not mask or enhance the effect of genotype, expression levels of 17q12-q21 genes were analyzed considering genotype alone as well as sex and genotype of the donor (Fig 3B and 3C). Both sex and genotype influenced expression of *ORMDL3* (*p<0*.*05* and *p<0*.*0001*, for sex and genotype, respectively, two-way ANOVA) and *ZPBP2* (p≤0.0005, for sex and genotype, two-way ANOVA) (Fig 3C). LCLs carrying rs11557467-G alleles tended to have higher RNA levels of *NR1D1* compared to rs11557467-T homozygotes, but these differences did not reach statistical significance (Fig 3B). No differences between males and females with respect to *NR1D1* levels were observed (Fig 3C).

### *Zpbp2* influences *Ormdl3* RNA levels in mouse liver

To determine if the *Zpbp2* region was involved in regulation of *Ormdl3* or *Nr1d1* expression in mice, we used a mouse strain that lacks *Zpbp2* and focused on lungs, small intestine (ileum) and liver, the three organs that are affected in human diseases with association to the *ZPBP2-ORMDL3* region, i.e. asthma, IBD and PBC.

**Fig 4:**
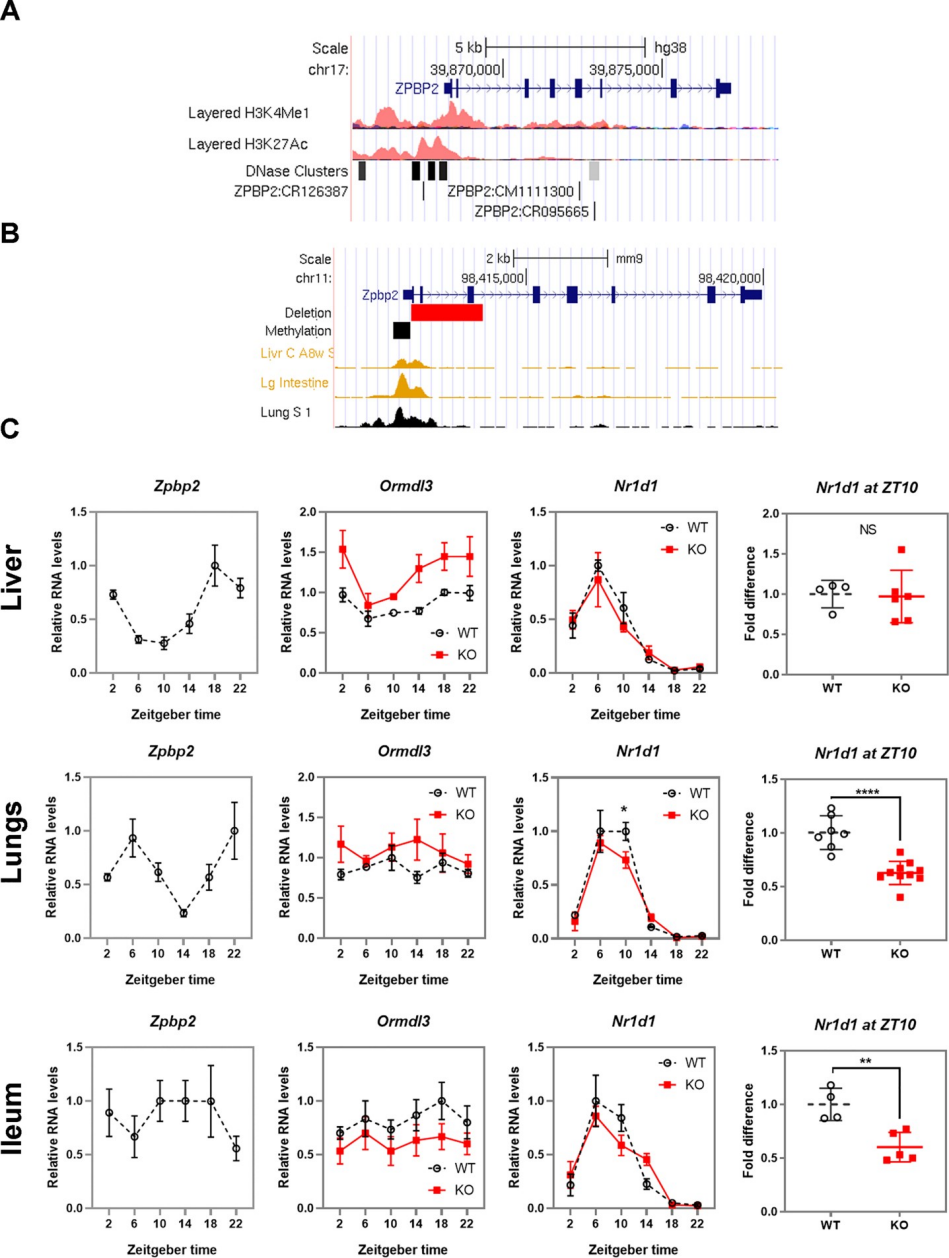
The *Zpbp2* deletion alters expression profiles of *Ormdl3* and *Nr1d1*. **A. The human *ZPBP2* region** harbors several regulatory features, i.e. enhancer marks H3K4me and H3K27ac, DNAse I hypersensitive sites, and regulatory polymorphisms that influence expression of *ORMDL3* and *GSDMB*. All features are shown in the context of the UCSC genome browser (hg38) (https://genome.ucsc.edu). **B. The mouse *Zpbp2* region**. The deletion (red box) and highly methylated region (black box) of the *Zpbp2* promoter overlap with DNAse I hypersensitivity sites in liver, lungs and small intestines (ileum) similar to the enrichment of enhancer marks in the human *ZPBP2* region. All features are shown in the context of the UCSC genome browser (mm9) (https://genome.ucsc.edu). **C. Diurnal profiles of gene expression levels in mouse liver, lungs, and ileum.** Expression levels of *Zpbp2*, *Ormdl3* and *Nr1d1* were determined in WT and KO mice at 6 time points (n = 3 per time point) and normalized to housekeeping gene *Eef2* and the expression maximum in WT mice was set to 1. Error bars for diurnal expression levels show standard error of the mean (SEM). *Nr1d1* expression differences between the KO and WT mice at ZT10 were validated in an independent set of mice and average expression in WT mice was set at 1. Error bars show standard deviation (SD). Significant differences in expression levels between WT and *Zpbp2* KO mice are indicated by asterisks * *p<0*.*05*, **p<0.*01*, **** p<0.*0001*.

The mouse *Zpbp2* region is orthologous to the human *ZPBP2* region that harbors the functional *cis*-regulatory SNPs rs4795397 and rs12936231 (Fig 4A and 4B). The *Zpbp2* knock-out mice (KO) carry a ~1.5 kb deletion of the *Zpbp2* exons 1 to 3 and do not express *Zpbp2* [29, 30]. The deletion removes part of the *Zpbp2* promoter and enhancer regions and is also associated with hypermethylation of the remainder of the *Zpbp2* promoter, DNAse I hypersensitive sites and putative enhancer (Fig 4B) [29].

In the liver of WT mice, expression levels of *Zpbp2* and *Ormdl3* varied over time with peak levels during the night (ZT18) and trough during the day (ZT6) (*p<0*.*0001* and *p<0*.*005*, respectively, one-way ANOVA) similar to the pattern observed in mice in other studies (Fig 2, S1 Fig) [41–43]. *Nr1d1* RNA levels peaked during the day (ZT6) and dropped during the night (ZT18), as expected (*p<0*.*0001*, one-way ANOVA).

If the deleted region harbored DNA elements that influenced *Nr1d1* regulation, we would observe differences in *Nr1d1* expression between the KO mice and WT controls. If the *Zpbp2* region was involved in the regulation of *Ormdl3*, as suggested by human data [10, 11], we would expect to find changes in *Ormdl3* levels. The *Zpbp2* deletion did not alter the liver *Nr1d1* expression profile. However, *Ormdl3* RNA levels were higher in the KO mice compared to controls (Fig 4C) (*p<0*.*005* and *p<0*.*0001*, for the effects of time and genotype, respectively, two-way ANOVA).

### The *Zpbp2* deletion is associated with changes in the regulation of *Nr1d1* in lungs and ileum

In lungs, *Zpbp2* levels changed with time (*p<0*.*05*, one-way ANOVA), whereas *Ormdl3* levels did not show time-dependent oscillations. Our WT mice had two *Zpbp2* peaks during the day. *Nr1d1* was expressed rhythmically with a peak during the day (ZT6-ZT10) and low expression at night (ZT18-ZT22). KO mice had lower *Nr1d1* expression at ZT10 (*p<0*.*05*, two-way ANOVA followed by multiple comparisons and Sidak’s correction for multiple testing) (Fig 4C). The effect of the deletion on ZT10 *Nr1d1* levels was confirmed in an independent set of WT and KO samples (*p<0*.*0001*, t-test statistics). KO mice also had a minor albeit statistically significant increase in *Ormdl3* RNA levels (***d*** = 0.73, *p<0*.*05*, for the effect of genotype, two-way ANOVA) (Fig 4C and S2 Fig**).**

In ileum, no circadian oscillations in expression of *Zpbp2* or *Ormdl3* were observed. KO mice had ~25% lower *Ormdl3* levels compared to WT mice (***d*** = 0.92, *p<0*.*05* for the effect of genotype, two-way ANOVA) (Fig 4C and S2 Fig**)**. *Nr1d1* levels were lower at ZT10 in KO mice compared to WT controls, similar to the decrease observed in lungs. Lower ZT10 *Nr1d1* levels in KO mice were confirmed in an independent set of mice (*p<0*.*005*, t-test statistics) (Fig 4C**)**.

### The *Zpbp2* deletion impacts the PPAR and cytokine signalling pathways in the mouse lung

**Fig 5:**
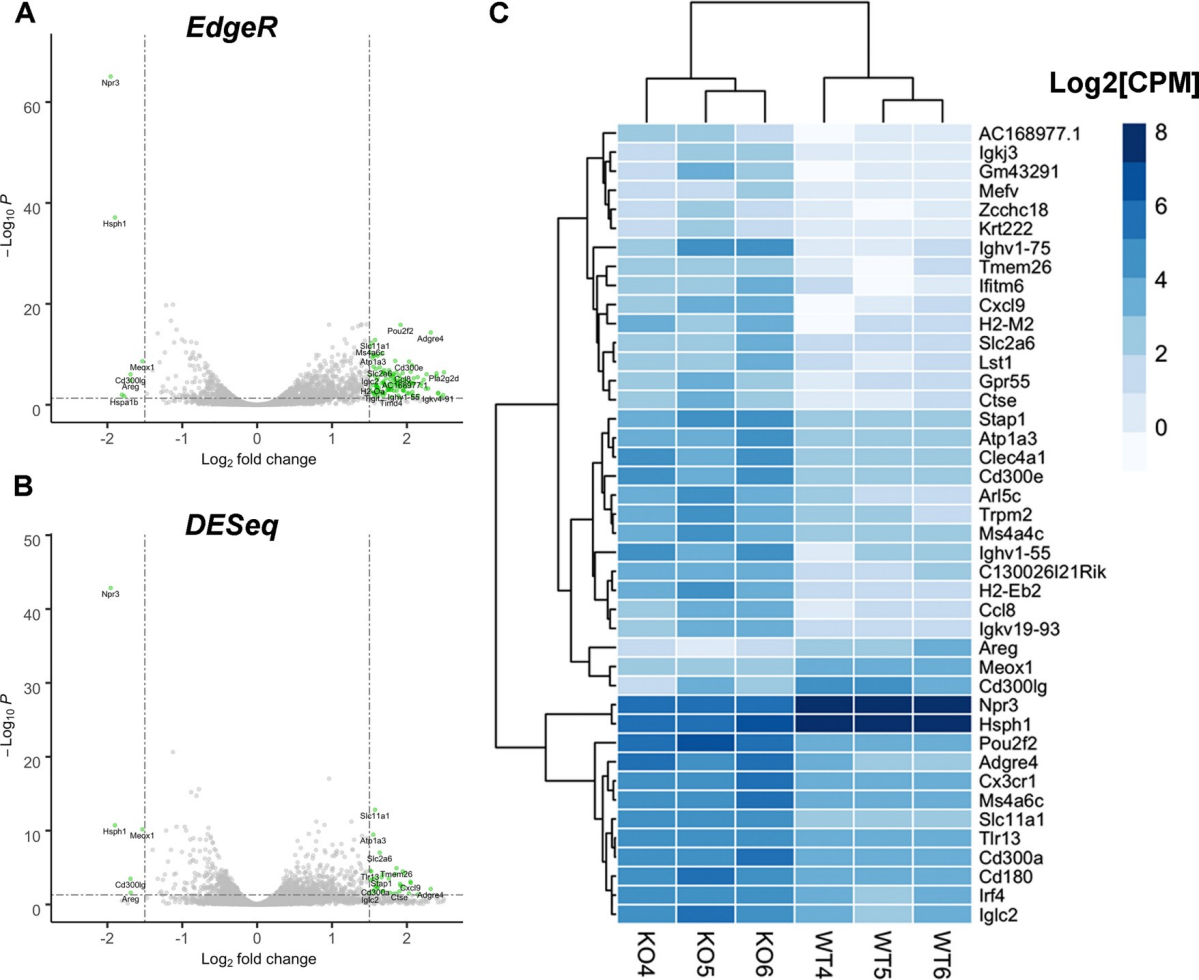
Deletion of *Zpbp2* influences the mouse lung transcriptome. **A. Differential expression analysis results using *EdgeR*.** Dotted lines mark the cut-offs at absolute fold change difference ≥1.5 and adjusted p-value ≤ 0.05. **B. Differential expression analysis results using *DESeq*.** Dotted lines mark the cut-offs at absolute fold change difference ≥1.5 and adjusted p-value ≤ 0.05. **C. Expression heatmap of the 42 differentially expressed genes.** Gene expression in Log_2_ Counts Per Million (CPM); a darker shade of blue indicates higher expression.

Deletion of *Zpbp2* changes the expression profile of the core clock gene *Nr1d1* in lungs. We therefore expected that differences between the lung transcriptomes of KO and WT mice would affect multiple genes and pathways regulated by the circadian clock. To identify the pathways affected by the *Zpbp2* deletion in lungs at two different time points, RNA-seq was performed. At ZT7, 40 differentially expressed genes (DEGs) with absolute fold difference |logFC| ≥1.5 and adjusted p-value ≤ 0.05 were identified and used for pathway analysis (S2 Table). At ZT10, 42 DEGs were identified and used for pathway analysis (Fig 5, S2 Table). The deletion influenced genes in the PPAR, adipocytokine and chemokine signalling pathways (Table 1).

**Table 1:** Biological pathways affected by the *Zpbp2* deletion in mouse lungs.

ZT7	ZT10
mmu03320: PPAR signaling pathway	mmu04062: Chemokine signaling pathway
mmu04920: Adipocytokine signaling pathway	mmu04060: Cytokine-cytokine receptor interaction
mmu04010: MAPK signaling pathway	
mmu04713: Circadian entrainment	
mmu04915: Estrogen signaling pathway	
mmu04152: AMPK signaling pathway	

## Discussion

We demonstrate that in the mouse *Zpbp2* and *Ormdl3* are regulated by the circadian clock in a tissue-specific fashion. Moreover, deletion of part of the mouse *Zpbp2* gene that removes the region orthologous to the human *cis*-regulatory region is associated with significant changes in the diurnal expression profile of the core clock gene *Nr1d1* in lungs and ileum, but not liver. *Nr1d1* encodes a transcription factor that influences regulation of thousands of genes throughout the genome [26, 45]. Therefore, by modifying *Nr1d1* expression the *Zpbp2* deletion affects expression of multiple genes in the lungs. At ZT7, PPAR signaling, which is known to play a critical role in aligning the circadian clock and lipid metabolism, is one of the most pronounced changes. This is particularly interesting since the *Zpbp2* KO mice are prone to obesity as they age and show changes in sphingolipid metabolism, i.e. lower levels of sphingosine-1-phosphate (S1P) and very long chain ceramides [29]. Reduced levels of S1P are consistent with the increase in the expression of *Ormdl3*, which is an inhibitor of sphingolipid metabolism. At ZT10, *Zpbp2* KO mice have higher expression levels of genes involved in chemokine signaling, inflammation and immunity, i.e. processes that are intertwined with circadian rhythms (reviewed in [24]). NR1D1 is a transcriptional repressor and controls cytokine production and inflammation [24, 46, 47]. It is therefore plausible that a modest decrease in *Nr1d1* levels in *Zpbp2* KO mice is associated with higher expression of several immunity-related genes, e.g. interferon regulatory factor 4 (*Irf4*), C-C motif chemokine ligand 8 (*Ccl8*), toll-like receptor 13 (*Tlr13*), C-X-C motif chemokine ligand 9 (*Cxcl9*), and chemokine (C-X3-C motif) receptor 1 (*Cx3cr1*). Several orthologs of genes implicated in susceptibility to inflammatory conditions in humans, e.g. solute carrier family 11 (proton-coupled divalent metal ion transporters), member 1 (*Slc11a1*, also known as *Nramp*) and amphiregulin (*Areg*) are among the ZT10 DEGs. It is also worth keeping in mind that we used rather stringent criteria for DEG selection. Including genes with smaller than 3-fold difference in expression levels dramatically increases the number of DEGs (Fig 5A and 5B).

Several lines of evidence suggest that the human *ZPBP2* region harbors an enhancer and a CTCF-binding site whose functions are modified by genetic variants associated with predisposition to asthma and several other chronic inflammatory diseases [10, 11]. Genetic variants in the *ZPBP2* gene are also associated with changes in the expression levels of *ORMDL3* in human cells [10, 11]. Our data support the role of the *ZPBP2* region in the regulation of *ORMDL3*, as deletion of the mouse orthologous region is associated with changes in *Ormdl3* RNA levels in mouse liver, lungs and ileum. Moreover, the deletion has opposite effects in liver and ileum, i.e. leads to upregulation of *Ormdl3* in liver and reduced expression in ileum.

Our data show that the *Zpbp2* region is involved in the regulation of *Nr1d1*. These findings are novel and demonstrate the importance of the inclusion of the time axis in GWAS follow-up studies, including eQTL analyses and mouse model-based experiments. Our data are consistent with published results that show chromatin interactions between the *Zpbp2* and *Nr1d1* genic regions [28] and data from 4C-seq experiments done by other groups that demonstrate interactions between the *Nr1d1* “super-enhancer” and the *Zpbp2* and *Ormdl3* regions [48]. This may explain why this deletion leads to changes in the diurnal expression profile of *Nr1d1*, which is located 200 kb away.

Remarkably, the *Zpbp2* deletion altered *Nr1d1* expression levels only at one of the tested time points, at ZT10. We speculate that such a time specificity points to a time-dependent interaction between the *Zpbp2* and *Nr1d1* genes. Interestingly, levels of the CLOCK protein and its association with chromatin peak at ZT10 [27, 49]. Given that *Nr1d1* transcription is tightly regulated by the CLOCK/BMAL1 complex [50], it is possible that the effect of the deletion on *Nr1d1* expression is mediated through changes in CLOCK binding. Whether the deletion changes the configuration of chromatin loops or it is the *Zpbp2* gene product that is critical for *Nr1d1* regulation will have to be determined in future studies.

To detect a time-specific change in the human, one would have to analyze *NR1D1* expression in both genotype- and time-dependent fashion in different cell types. Therefore, we speculate that lack of a significant *cis*-effect of the *ZPBP2* rs11557467 genotype on *NR1D1* expression in human LCLs as shown here as well as in GTEX data [51] is insufficient to refute the hypothesis of regulatory interactions between the two genes in human cells and that genetic variants influence the *NR1D1* expression profile.

Our recent studies found interaction between 17q12-q21 alleles, DNA methylation levels, sex, and asthma with genetic association being more pronounced in males and DNA methylation of the *ZPBP2* promoter region being associated with reduced risk of asthma in females [52, 53]. Therefore, we asked if sex was also a modifier of gene expression levels. In human LCLs, we observed a sex bias in the levels of *ORMDL3* (higher in females) and *ZPBP2* (higher in males), but not *NR1D1*. In mice, no evidence for sex bias in the regulation of *Ormdl3*, *Zpbp2*, or *Nr1d1* was found ([29] and present study).

The ensemble of published works suggests that daVs and variation in DNA methylation influence transcriptional regulation of several genes residing in the 17q12-q21 region with *ORMDL3* and *GSDMB* having been investigated in greater detail as potential disease gene candidates (reviewed in [54]). Based on our new data, we propose that *NR1D1* should be added to the list of gene candidates underlying the GWAS associations in the *ZPBP2-ORMDL3* region. Further studies in humans are necessary to test this hypothesis and clarify the importance of diurnal variation for the genetic association between 17q12-q21 alleles and disease.

## Supporting information

S1 Fig **Diurnal oscillations in the expression levels of *Nr1d1*, *Ormdl3*, and *Zpbp2*** in the (A) distal colon and (B) liver of mice housed in LD (light/dark) conditions (data from [42, 43]).
(TIF)

S2 Fig *Ormdl3* expression levels in KO and WT mice in different organs. The y-axis shows *Ormdl3* RNA levels normalized by *Eef2*. Error bars represent SD. Significant differences in expression levels between WT and *Zpbp2* KO mice are indicated by asterisks * *p<0*.*05*, **** p<0.*0001*.
(TIF)

S1 Table Primers used for qPCR experiments (5’- 3’). (DOCX)

S2 Table List of lung differentially expressed genes and transcripts. (DOCX)
