# Supplementary figures and images for: Regulatory interaction between the ZPBP2-ORMDL3/Zpbp2-Ormdl3 region and the circadian clock
Source: PLoS One. 2019 Sep 27;14(9):e0223212. doi: 10.1371/journal.pone.0223212 (PMC6764692; doi:10.1371/journal.pone.0223212)

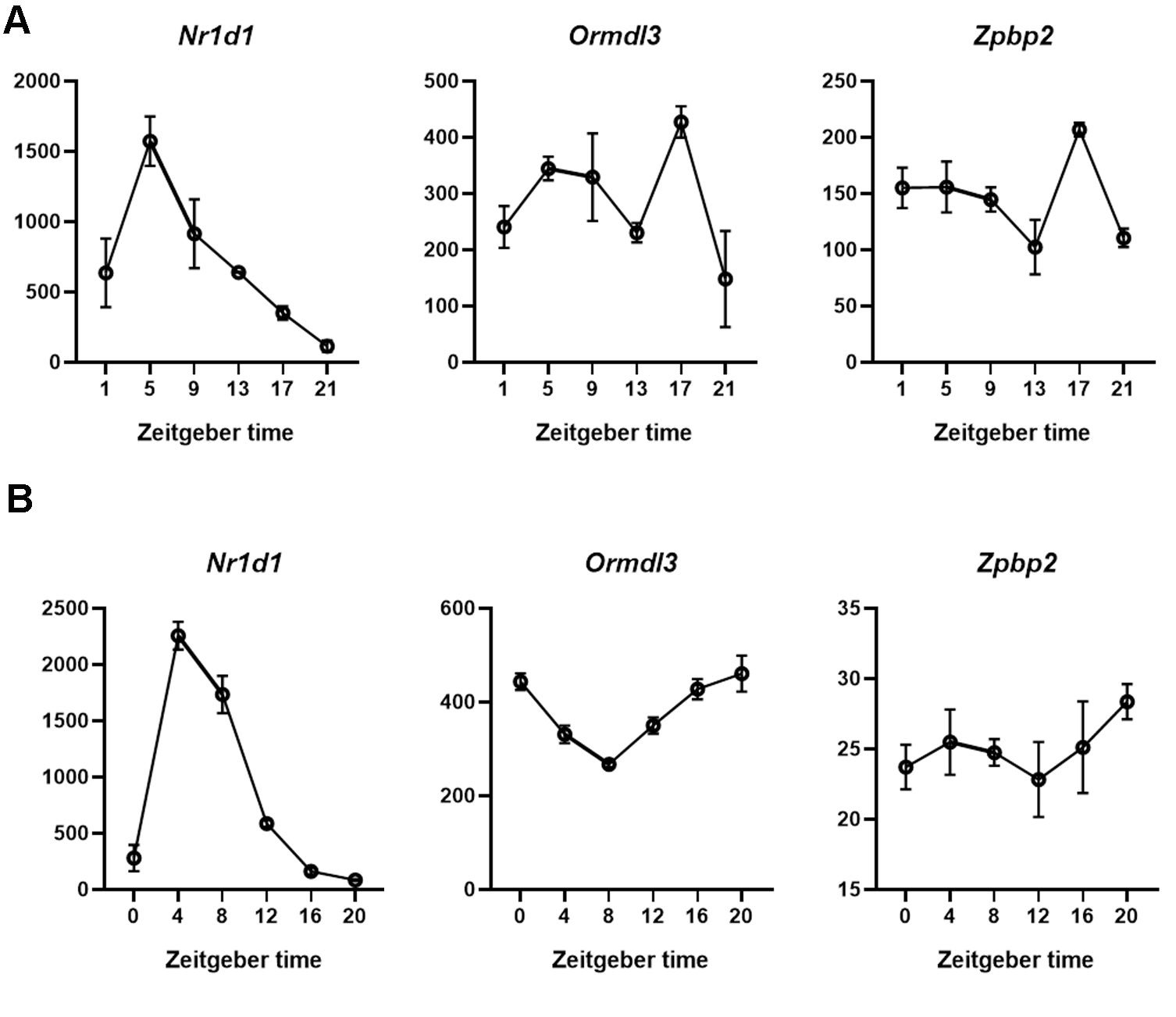

Supplement: S1 Fig — Diurnal oscillations in the expression levels of Nr1d1, Ormdl3, and Zpbp2 in the (A) distal colon and (B) liver of mice housed in LD (light/dark) conditions (data from [42, 43]). (TIF) [file pone.0223212.s001.tif]

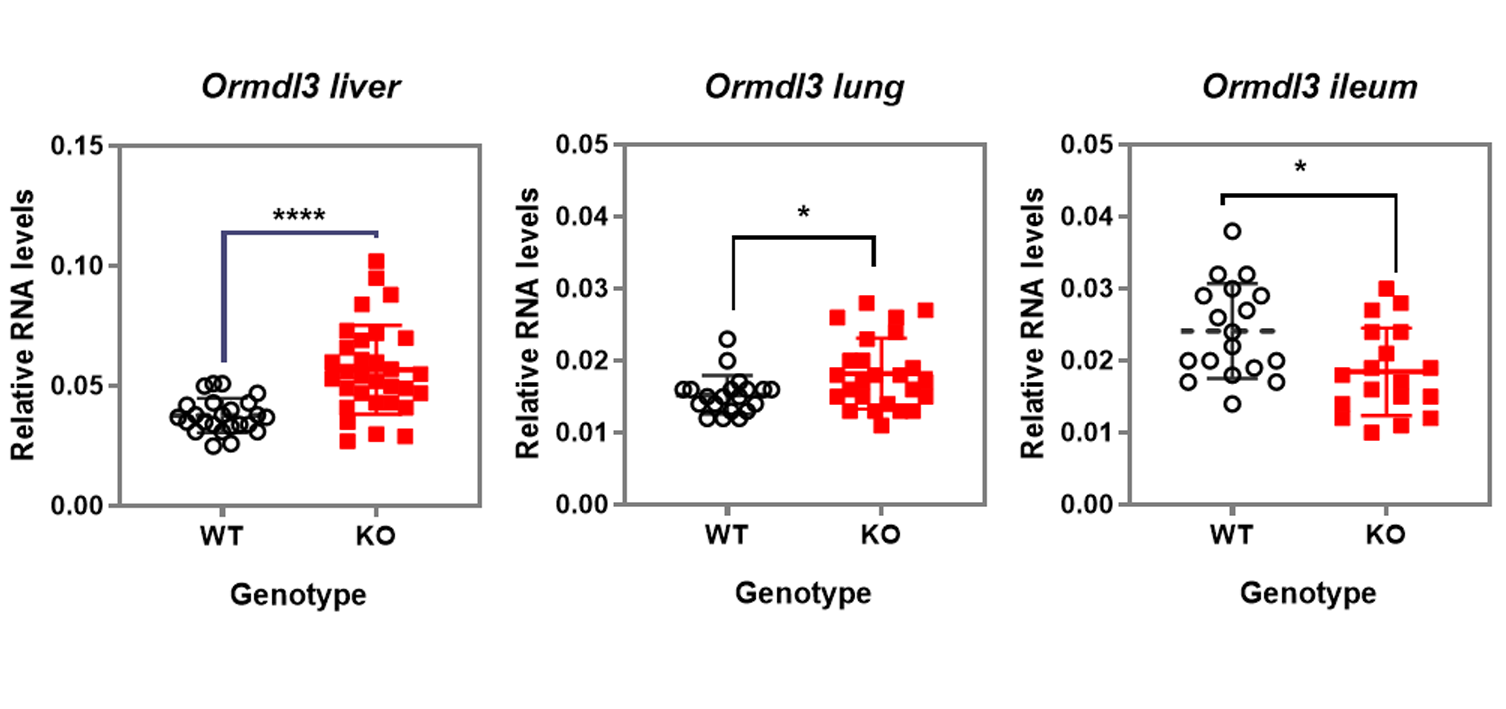

Supplement: S2 Fig — The y-axis shows Ormdl3 RNA levels normalized by Eef2. Error bars represent SD. Significant differences in expression levels between WT and Zpbp2 KO mice are indicated by asterisks * p<0.05, **** p<0.0001. (TIF) [file pone.0223212.s002.tif]
