# Supplementary material for: Regulatory interaction between the ZPBP2-ORMDL3/Zpbp2-Ormdl3 region and the circadian clock
Source: PLoS One. 2019 Sep 27;14(9):e0223212. doi: 10.1371/journal.pone.0223212 (PMC6764692; doi:10.1371/journal.pone.0223212)
Supplement: S1 Table — (DOCX) [file pone.0223212.s003.docx]

**S1 Table.** **Primers used for qPCR experiments (5’- 3’)**

| Gene | Forward | Reverse |
| --- | --- | --- |
| *Nr1d1* | GCGGTGGTGCTTGTCTCT | TTCTCGGAATGCATGTTGTT |
| *Ormdl3* | AGACTCCAGACCAAGGCAAA | GACAGGTGACATCCACATCG |
| *Zpbp2* | TTTTCGGAAACAAGCACACA | GACAGGTGACATCCACATCG |
| *Eef2* | GCTGGTACCCCCATGTTTG | CGCGTCTCAGCTACCACTT |
