# Supplementary material for: Regulatory interaction between the ZPBP2-ORMDL3/Zpbp2-Ormdl3 region and the circadian clock
Source: PLoS One. 2019 Sep 27;14(9):e0223212. doi: 10.1371/journal.pone.0223212 (PMC6764692; doi:10.1371/journal.pone.0223212)
Supplement: S2 Table — (DOCX) [file pone.0223212.s004.docx]

**S2 Table. List of lung differentially expressed genes and transcripts.**

| ZT7 | | ZT10 | |
| --- | --- | --- | --- |
| Gene Symbol | **LogFC (KO vs WT)** | **Gene Symbol** | **LogFC (KO vs WT)** |
| *Bmp10* | -12.9 | *Npr3* | -2.0 |
| *Ucp1* | -9.1 | *Hsph1* | -1.9 |
| *Myl2* | -6.6 | *Cd300lg* | -1.7 |
| *Pck1* | -3.7 | *Areg* | -1.7 |
| *Lep* | -3.2 | *Meox1* | -1.5 |
| *Tmem45b* | -3.1 | *Tlr13* | 1.5 |
| *Plin1* | -3.1 | *Iglc2* | 1.5 |
| *A530016L24Rik* | -2.9 | *Ms4a6c* | 1.5 |
| *Alpk2* | -2.9 | *Cx3cr1* | 1.5 |
| *Thrsp* | -2.8 | *Irf4* | 1.5 |
| *Corin* | -2.6 | *Atp1a3* | 1.6 |
| *Pnpla3* | -2.5 | *Clec4a1* | 1.6 |
| *Adipoq* | -2.4 | *Ms4a4c* | 1.6 |
| *Cd300lg* | -2.3 | *Slc11a1* | 1.6 |
| *Fabp4* | -2.3 | *Cd300a* | 1.6 |
| *Apol6* | -2.2 | *Trpm2* | 1.6 |
| *Mrap* | -2.2 | *Lst1* | 1.6 |
| *Cacna1h* | -2.2 | *Gpr55* | 1.6 |
| *Aoc3* | -2.1 | *Cd180* | 1.6 |
| *Ryr2* | -2.0 | *Slc2a6* | 1.6 |
| *Plin4* | -2.0 | *Stap1* | 1.7 |
| *Car3* | -1.8 | *Zcchc18* | 1.7 |
| *Cidec* | -1.7 | *C130026I21Rik* | 1.7 |
| *Sncg* | -1.7 | *Igkj3* | 1.7 |
| *Casq2* | -1.7 | *Mefv* | 1.8 |
| *Ndrg4* | -1.7 | *Igkv19-93* | 1.8 |
| *Tenm4* | -1.7 | *H2-Eb2* | 1.8 |
| *Nat8l* | -1.6 | *Arl5c* | 1.8 |
| *Dgat2* | -1.5 | *Krt222* | 1.9 |
| *Pdk4* | -1.5 | *Tmem26* | 1.9 |
| *Ckmt2* | -1.5 | *Ctse* | 1.9 |
| *Zfp36* | 1.6 | *H2-M2* | 1.9 |
| *Cyr61* | 1.8 | *Pou2f2* | 1.9 |
| *Junb* | 1.8 | *Ccl8* | 1.9 |
| *Fos* | 2.6 | *Ighv1-55* | 2.0 |
| *Gm26917* | 3.3 | *AC168977.1* | 2.0 |
| *Hspa1a* | 3.4 | *Cd300e* | 2.0 |
| *Hspa1b* | 3.4 | *Cxcl9* | 2.0 |
| *Alb* | 4.0 | *Ifitm6* | 2.1 |
| *BC018473* | 8.7 | *Gm43291* | 2.1 |
|  |  | *Adgre4* | 2.3 |
|  |  | *Ighv1-75* | 3.5 |
